# MicroRNA-183-3p Is a Predictor of Worsening Heart Failure in Adult Patients With Transposition of the Great Arteries and a Systemic Right Ventricle

**DOI:** 10.3389/fcvm.2021.730364

**Published:** 2021-09-08

**Authors:** Masood Abu-Halima, Eckart Meese, Hashim Abdul-Khaliq, Tanja Raedle-Hurst

**Affiliations:** ^1^Institute of Human Genetics, Saarland University Medical Center, Homburg, Germany; ^2^Department of Pediatric Cardiology, Saarland University Medical Center, Homburg, Germany

**Keywords:** microRNA, transposition of the great arteries, heart failure, systemic right ventricle, biomarker

## Abstract

**Aim:** MicroRNAs (miRNAs) have been shown to play an important role in the progression of heart failure (HF). The aim of our study was to analyze miRNAs in the blood of patients with transposition of the great arteries and a systemic right ventricle (TGA-RV) in order to identify those that predict worsening HF.

**Materials and Methods:** In 36 patients with TGA-RV, SurePrint™ 8 × 60K Human v21 miRNA microarrays were used to determine the miRNA abundance profiles and compared to 35 age- and gender-matched healthy volunteers (HVs). MiRNAs that were most significantly abundant or best related to worsening HF were further validated by RT-qPCR.

**Results:** Using miRNA array analysis, a total of 50 down-regulated and 56 up-regulated miRNAs were found to be differentially abundant in TGA-RV patients compared to HVs. Six of these 106 miRNAs were significantly related to worsening HF. After validation by RT-qPCR, four miRNAs turned out to be significantly associated with worsening HF, namely miR-150-5p, miR-1255b-5p, miR-423-3p, and miR-183-3p. In the stepwise multivariable Cox regression analysis, ejection fraction of the systemic RV, high sensitive TNT and miR-183-3p were found to be independent predictors of worsening HF (*P* = 0.001, *P* = 0.002, and *P* = 0.001, respectively).

**Conclusions:** In patients with TGA-RV, miR-183-3p is an independent predictor of worsening HF and thus may be used as additional biomarker in the risk assessment of these patients.

## Introduction

Transposition of the great arteries (TGA) is a rare congenital heart defect accounting for 5–7% of all congenital heart abnormalities (1). The most common form is complete TGA, also known as d-TGA, in which the blood flow runs in two parallel circuits and thus usually is associated with severe cyanosis. Since complete TGA is a cyanotic heart defect, early correction of the pathological blood flow is mandatory. In former times, an atrial switch procedure had been performed redirecting blood flow on the atrial level using baffles. However, this surgical method implies a morphologically right ventricle to be in the systemic position. Although the atrial switch procedure has been replaced by the arterial switch procedure nowadays, there are still a lot of patients suffering from the sequelae after the atrial switch i.e., atrial arrhythmias and heart failure (2). Another and more rare form is congenitally corrected TGA (ccTGA) that is associated with physiologically normal blood flow due to ventricular inversion but also carries the risk of heart failure because the systemic ventricle is also a morphologically right ventricle. In patients with congenital heart disease (CHD), those with a systemic morphological right ventricle or univentricular heart are known to be at risk for heart failure (HF) (3–5). Moreover, the occurrence of overt HF also has negative implications on the prognosis of CHD patients (6, 7). As a result, prevention or early treatment of HF is crucial in order to avoid worsening of HF that is associated with a poor prognosis in these patients (8). MicroRNAs (miRNAs) are a class of genome-encoded small RNAs (18–22 nucleotides) that serve as important regulators of gene expression via a sequence-specific interaction with the 3' untranslated region (3'UTR) of the messenger RNA (mRNA), thus decreasing the stability or inhibiting the translation of mRNA (9, 10). Currently, there are 2,300 “real” miRNAs in the miRNA database based on miRBase.org (11). It is assumed, that almost every cellular and biological process is regulated by miRNAs, including processes regulating CHD (12–19). MiRNAs are also known to play an important role in the onset and progression of HF (20–22). Since they are also disease-specific, miRNA signatures associated with HF may vary according to the underlying heart abnormality. In patients with complex CHD, specific miRNAs have been found to be associated with the presence of symptomatic HF or even have additional prognostic value to natriuretic peptides (14, 15). With respect to TGA patients, there are two studies investigating miRNAs. In the study by Lai et al. 11 miRNAs were validated to be up-regulated in TGA patients after atrial switch operation of which miR-18a and miR-486-5p were negatively related to the contractility of the systemic right ventricle as assessed by myocardial acceleration during isovolumic contraction (IVA) derived from tissue Doppler imaging (23). In another study by Tutarel et al. miR-423-5p failed to be associated with the systolic function of the systemic right ventricle as assessed by the ejection fraction via magnetic resonance imaging in TGA patients after atrial switch procedure (24). However, this negative result may be due to the fact that patients had only mild heart failure symptoms in that study and that the discriminative power of miR-423-5p was limited due to the lack of advanced stages of HF in the study cohort. Indeed, in another study, miR-423-5p has been identified as a reliable biomarker for left heart failure including patients with severe stages of HF (25). On the other hand, it is known that miRNA patterns may be different in right and left ventricular chambers and that the two chambers also display different responses under pathological conditions (20). In patients with TGA and a systemic right ventricle (TGA-RV), the hemodynamic situation is even more complicated since a right ventricle has to serve the systemic circulation and deal with higher systemic pressure and resistance resulting in maladaptive remodeling. The aim of our study, therefore, was to analyse miRNA signatures in TGA-RV patients in order to identify those miRNAs that are associated with worsening HF and thus may yield prognostic impact.

## Materials and Methods

### Patients

In our outpatient clinic for adult CHD, a total of 36 consecutive patients with TGA and a systemic right ventricle (TGA-RV) were enrolled in the present study of whom 26 patients had d-TGA after atrial switch operation and 11 patients congenitally corrected TGA (ccTGA). The recruitment of patients and healthy volunteers (HVs) comprised the period from April 2015 until December 2019. Baseline characteristics of patients are shown in Table 1.

**Table 1:** Characteristics of TGA-RV patients according to the underlying heart defect.

**Variables**	**All patients** **(*n* = 36)**	**d-TGA after atrial switch** **(*n* = 25)**	**ccTGA** **(*n* = 11)**	***P-*value**
Age at follow-up (years)	36.3 ± 12.3	31.6 ± 6.3	47.0 ± 15.9	0.006
Male sex	25/36 (69.4%)	20/25 (80%)	5/11 (45.5%)	0.056
Patients with worsening HF	8/36 (22.2%)	6/25 (24%)	2/11 (18.2%)	1.000
Patients with the occurrence of recurrent atrial arrhythmias or presence of atrial fibrillation	11/36 (30.6%)	7/25 (28%)	4/11 (36.4%)	0.703
NYHA functional class	1.5 ± 0.7	1.4 ± 0.6	1.7 ± 0.8	0.216
Systolic blood pressure (mmHg)	127.6 ± 14.1	128.8 ± 10.9	124.9 ± 20.0	0.186
Diastolic blood pressure (mmHg)	73.1 ± 10.1	74.2 ± 9.2	70.6 ± 12.0	0.209
Transcutaneous oxygen saturation at rest (%)	96.8 ± 1.8	96.7 ± 1.9	97.1 ± 1.8	0.464
Ejection fraction of systemic RV (%)	48.6 ± 11.6	49.0 ± 11.5	47.5 ± 12.1	0.770
End-diastolic volume of systemic RV (ml)	135.4 ± 61.2	143.6 ± 68.0	116.6 ± 38.2	0.345
End-systolic volume of systemic RV (ml)	73.5 ± 54.0	77.9 ± 61.0	63.5 ± 33.2	0.536
VTI above aortic valve (cm)	23.4 ± 4.2	23.4 ± 3.9	23.6 ± 5.1	0.668
Albumin (g/l)	47.0 (45.3–49.0)	47.0 (46.0–50.5)	45.0 (42.0–48.0)	0.009
Creatinine (mg/dl)	0.90 (0.76–1.00)	0.91 (0.79–1.00)	0.87 (0.75–0.99)	0.582
eGFR (ml/min)	106.1 (90.9–112.7)	107.3 (96.2–114.5)	94.2 (69.1–108.1)	0.012
NT-proBNP (pg/ml)	244.4 (163.5–469.5)	234.2 (131.0–468.5)	313.7 (237.5–472.6)	0.096
High sensitive troponin T (pg/ml)	7.0 (5.0–11.8)	7.0 (5.0–8.0)	11.0 (4.0–15.0)	0.247

*NYHA, New York Heart Association; HF, heart failure; VTI, velocity-time integral; eGFR, estimated glomerular filtration rate*.

*Mean ± standard deviation or median (interquartile interval) are used*.

*d-TGA compared to ccTGA subgroup*.

All patients were regularly seen in our clinic on a 1-year basis and underwent the same study protocol that has been described in detail previously (15) including 12-lead electrocardiogram, transthoracic two-dimensional echocardiography, and laboratory tests. The study was conducted prospectively; mean follow-up time was 37.2 ± 20.7 months. During follow-up, 8/36 (22.2%) patients experienced worsening HF that was defined as a rise of NT-proBNP levels >1,000 pg/ml or the occurrence of overt HF, i.e., clinical signs of acute HF. Worsening HF was associated with recurrent atrial arrhythmias in 3/8 patients and the presence of atrial fibrillation in 2/8 patients. A total of 11/36 (30.6%) patients presented with atrial arrhythmias that required electrical cardioversion or antiarrhythmic drug therapy but was not associated with worsening HF in 6/11 (54.5%) patients. For the identification of abundant miRNAs, 35 HVs served as controls. All HVs were selected in an age- and sex-matched manner and underwent the same study protocol as the patients to rule out any structural heart abnormality. The control group was mainly recruited at our institution and consisted of medical staff as well as medical students; however, to avoid selection bias, individuals of other, non-medical institutions were also enrolled. The study complies with the declaration of Helsinki and good clinical practice guidelines. It was approved by the ethical board of the Saarland Medical Association. All patients and HVs gave written informed consent before enrollment into the study.

### Sample Preparation, RNA Isolation, and Quality Assessments

In all patients and HVs, blood samples for miRNA detection were drawn in PAXgene™ blood tubes (Becton–Dickinson, Heidelberg, Germany) shortly after echocardiographic evaluation. PAXgene™ collected blood was kept at room temperature for 24 h to ensure complete lysis of the blood cells, stored at −20°C for several days, and finally transferred to −80°C for long-term storage until RNA isolation. Total RNAs including miRNAs were purified from PAXgene™ samples using PAXgene™ Blood miRNA Kit on the QIAcube™ robot (Qiagen, Hilden, Germany) following the manufacturer's recommendations, and DNase I treatment step was included to eliminate the residual genomic DNA (Qiagen). The RNA yield and purity were determined using Agilent 2100 Bioanalyzer (Agilent Technologies, Santa Clara, CA, United States) and NanoDrop 2000 Spectrophotometer (ThermoFisher Scientific, Waltham, MA, United States). All RNA samples passed quality control with an RNA integrity number of ≥7.5.

### Analysis of miRNAs by Microarray

The abundance level of miRNA was carried out using the purified miRNA fraction from patients with TGA and HVs using SurePrint™ 8X60K Human v21 miRNA microarrays (Agilent Technologies) according to the manufacturer's instructions with slight modification. Briefly, 125 ng of extracted RNA including miRNAs from each sample was labeled, hybridized to the miRNA microarray chip, washed and the images were acquired using an Agilent DNA microarray scanner (Agilent Technologies). Finally, the Feature Extraction Software (Agilent Technologies) was used to extract miRNA abundance data.

### Microarray Data Analysis

Using Agilent feature extraction software (Agilent Technologies), the scanned microarray images were analyzed and processed in order to obtain background-subtracted, and outlier rejected signal intensities. The generated raw signal intensities were exported to the GeneSpring GX software (version 14.9.1, Agilent Technologies). Microarray raw signal intensities were normalized using quantile normalization and the differential abundant levels were identified for each sample. Fold change for the patients with TGA and HVs was obtained. Unpaired Student's *t*-test with False Discovery Rate (FDR) was applied, and the *P-*value was calculated based on the volcano plot algorithm (GeneSpring GX software). Consequently, the lower and higher abundant miRNAs were identified.

### Reverse Transcription and Quantitative Real-Time PCR (RT-qPCR) of miRNA

The abundance level of circulating miRNAs was quantified by RT-qPCR using the Biomark HD™ System (Fluidigm Corporation, California, United States) and the TaqMan™ microRNA Assays (Thermo Fisher Scientific) as previously described (26, 27). Briefly, complementary DNA (cDNA) was generated in 8 μL reactions by reverse transcription of 75 ng total RNA using the TaqMan® MicroRNA Reverse Transcription Kit and RT Primers Pool (10X) (Thermo Fisher Scientific). Following reverse transcription, 2.5 μL of the generated cDNA was preamplified by mixing 12.5 μL of TaqMan™ PreAmp Master Mix (2X) and 3.75 μL of PreAmp Primers Pool (10X) (Thermo Fisher Scientific) in 25 μL reaction volume. Following the preamplification of the cDNA, RT-qPCR was carried out with 96.96 Dynamic Array™ IFC for Gene Expression arrays (Fluidigm Corporation) as indicated in Fluidigm's protocol (PN 68000130 E1). Briefly, every 10X Assays contained 3 μL TaqMan Primer Assay (20X) (a mixture of forward and reverse primers, and probe) (Thermo Fisher Scientific), and 3 μL Assay Loading Reagent (2X) (Fluidigm, PN 85000736). Sample Pre-Mix was prepared by combining 3 μL TaqMan™ Universal PCR Master Mix, no AmpErase™ UNG (2X) (Thermo Fisher Scientific), 0.3 μL GE Sample Loading Reagent (20X) (Fluidigm, PN 85000735), and 2.7 μL pre-amplified cDNA for each sample. Finally, 5 μL of each Assay and Sample Mix were transferred into the appropriate inlets according to the Fluidigm's recommendation. After loading, the array was placed in the Biomark HD instrument for quantification and detection using GT 96 x 96 Standard v1 PCR thermal protocol. The data were analyzed with Real-Time PCR Analysis Software (Fluidigm Corporation) according to Fluidigm's recommendation. Negative control samples (H_2_O) were included in the reverse transcription, preamplification, and amplification steps and were finally defined as those with Ct values ≥35 or undetermined.

### Statistical Analysis

The statistical analysis was performed using the IBM SPSS Statistics program 25.0.3 (SPSS, Inc., Chicago, IL). Clinical variables are presented as mean ± standard deviation or median (interquartile interval) as appropriate. To test for differences between unpaired groups, a Mann–Whitney-*U* test was used for continuous variables and a chi-square test (or Fisher exact test, if numbers were small) for nominal variables. Spearman rank correlation was used to evaluate associations between variables. Cox regression analysis was performed in a univariable and multivariable model to identify predictors of worsening HF. For the multivariable model, all variables were entered that gave significant results in the univariable model applying a *P* < 0.1. A stepwise forward Cox regression analysis was performed to identify independent predictors of worsening HF. In general, a two-tailed *P* < 0.05 was considered statistically significant. The ΔCt (cycle threshold) was used to measure the dynamic change of specifically selected miRNAs using RNU6B small nuclear RNA (snRNA) as an endogenous reference miRNA as previously validated for this type of sample (12–17, 19, 28). MiRNAs were considered as differentially abundant if they obtained an adjusted *P* < 0.05 after applying the unpaired Student's *t*-test.

## Results

### Selection of Differentially Expressed miRNAs Using Microarray

**Figure 1:**
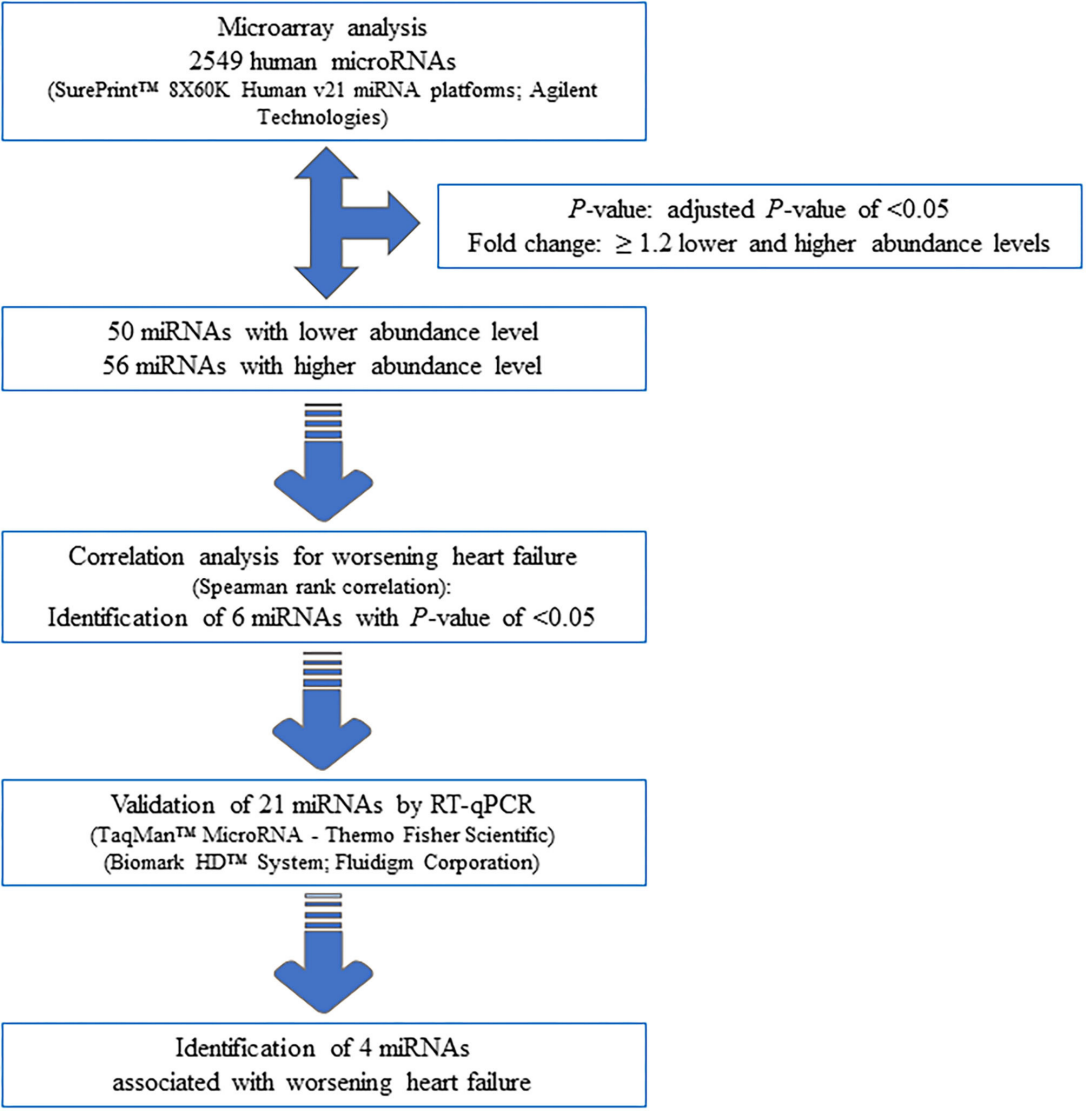
Workflow of the analysis for the identification of miRNAs associated with worsening HF.

Seventy-one samples (36 TGA-RV patients and 35 HVs) passed the miRNA microarray quality check. A flow chart representing the algorithm for miRNA selection is displayed in Figure 1. By considering only the miRNAs with an adjusted *P* < 0.05 and a fold change of ≥1.2 (i.e., lower and higher abundance levels) between the patient and control group, 106 miRNAs showed differentially altered abundance levels in TGA-RV patients as compared to HVs (Table 2). Of these 106 differentially abundant miRNAs, 50 miRNAs were significantly lower and 56 miRNAs were significantly higher in the abundance levels. All abundant miRNAs were related to parameters of worsening HF identifying 6 miRNAs that showed statistically significant associations (Table 3).

**Table 2:** Significantly abundant miRNAs in the blood of TGA-RV patients compared to HVs as determined by microarray.

**MicroRNA** **([TGA-RV] vs. [HVs])**	**Fold change**	**Regulation**	**Adjusted** ***P-*value**	**MicroRNA** **([TGA-RV] vs. [HVs])**	**Fold change**	**Regulation**	**Adjusted *P-*value**
hsa-miR-133b	3.96	Lower	0.00433	hsa-miR-6507-3p	3.98	Higher	0.00212
hsa-miR-4659a-3p	3.64	Lower	0.00008	hsa-let-7b-3p	3.73	Higher	0.00272
hsa-miR-1255b-5p	3.54	Lower	0.00122	hsa-miR-140-5p	3.47	Higher	0.02153
hsa-miR-1271-5p	3.32	Lower	0.00122	hsa-miR-590-5p	3.10	Higher	0.03269
hsa-miR-5690	3.18	Lower	0.00231	hsa-let-7f-1-3p	3.03	Higher	0.00962
hsa-miR-3200-3p	3.09	Lower	0.00285	hsa-miR-483-3p	3.01	Higher	0.00288
hsa-miR-7107-5p	3.08	Lower	0.00645	hsa-miR-17-3p	2.99	Higher	0.00930
hsa-miR-4485-3p	2.74	Lower	0.00233	hsa-miR-6865-3p	2.87	Higher	0.00836
hsa-miR-183-3p	2.68	Lower	0.00288	hsa-miR-425-3p	2.65	Higher	0.02353
hsa-miR-146b-5p	2.51	Lower	0.02811	hsa-miR-7114-5p	2.64	Higher	0.03429
hsa-miR-4746-3p	2.49	Lower	0.00256	hsa-miR-3150b-5p	2.57	Higher	0.01546
hsa-miR-4478	2.49	Lower	0.01014	hsa-miR-1825	2.53	Higher	0.03098
hsa-miR-193b-3p	2.46	Lower	0.01060	hsa-miR-6877-3p	2.51	Higher	0.00962
hsa-miR-550b-2-5p	2.43	Lower	0.00836	hsa-miR-301a-3p	2.48	Higher	0.03485
hsa-miR-181b-5p	2.30	Lower	0.02633	hsa-miR-4640-3p	2.47	Higher	0.01667
hsa-miR-942-3p	2.30	Lower	0.02444	hsa-miR-4725-5p	2.45	Higher	0.03245
hsa-miR-6513-3p	2.23	Lower	0.03456	hsa-miR-3646	2.43	Higher	0.01550
hsa-miR-3679-5p	2.21	Lower	0.02633	hsa-miR-6776-3p	2.41	Higher	0.00844
hsa-miR-99a-5p	2.21	Lower	0.00419	hsa-miR-6841-3p	2.41	Higher	0.00491
hsa-miR-6510-5p	2.19	Lower	0.03485	hsa-miR-1470	2.40	Higher	0.01240
hsa-miR-6891-5p	2.18	Lower	0.02444	hsa-miR-191-3p	2.18	Higher	0.01080
hsa-miR-3605-3p	2.18	Lower	0.02150	hsa-miR-6890-3p	2.14	Higher	0.04961
hsa-miR-1285-3p	2.16	Lower	0.00657	hsa-miR-6728-3p	2.12	Higher	0.01247
hsa-miR-5001-5p	2.16	Lower	0.03485	hsa-miR-6796-3p	2.11	Higher	0.02505
hsa-miR-10a-5p	2.07	Lower	0.04401	hsa-miR-1238-3p	2.10	Higher	0.01474
hsa-miR-330-3p	2.03	Lower	0.03934	hsa-miR-7977	2.07	Higher	0.00335
hsa-miR-7-1-3p	2.03	Lower	0.03485	hsa-miR-6889-3p	2.07	Higher	0.01667
hsa-miR-139-5p	2.02	Lower	0.03485	hsa-miR-6870-3p	2.01	Higher	0.03485
hsa-miR-5695	2.01	Lower	0.02904	hsa-miR-4433a-5p	1.98	Higher	0.03485
hsa-miR-8485	2.00	Lower	0.03789	hsa-miR-6508-5p	1.96	Higher	0.02260
hsa-miR-491-5p	1.99	Lower	0.01146	hsa-miR-6891-3p	1.92	Higher	0.03456
hsa-miR-1275	1.98	Lower	0.01474	hsa-miR-4667-3p	1.86	Higher	0.02444
hsa-miR-4659b-3p	1.79	Lower	0.03485	hsa-miR-19a-3p	1.79	Higher	0.00288
hsa-miR-148b-5p	1.79	Lower	0.01546	hsa-miR-142-3p	1.76	Higher	0.03204
hsa-miR-103a-2-5p	1.72	Lower	0.04961	hsa-miR-6819-3p	1.74	Higher	0.01606
hsa-miR-132-3p	1.64	Lower	0.00773	hsa-miR-6810-3p	1.70	Higher	0.04463
hsa-miR-3200-5p	1.61	Lower	0.03245	hsa-miR-939-3p	1.69	Higher	0.03514
hsa-miR-629-3p	1.60	Lower	0.02694	hsa-miR-6799-3p	1.62	Higher	0.03485
hsa-miR-454-5p	1.57	Lower	0.02911	hsa-miR-4649-3p	1.58	Higher	0.02987
hsa-miR-191-5p	1.54	Lower	0.00288	hsa-miR-1281	1.57	Higher	0.04397
hsa-miR-942-5p	1.40	Lower	0.02164	hsa-miR-106b-5p	1.54	Higher	0.00122
hsa-miR-423-3p	1.39	Lower	0.02444	hsa-miR-142-5p	1.53	Higher	0.02633
hsa-miR-574-3p	1.36	Lower	0.00288	hsa-miR-19b-3p	1.51	Higher	0.02444
hsa-miR-150-5p	1.35	Lower	0.00614	hsa-miR-29b-3p	1.49	Higher	0.02789
hsa-miR-342-3p	1.32	Lower	0.01146	hsa-miR-7975	1.47	Higher	0.02003
hsa-miR-128-3p	1.30	Lower	0.00836	hsa-miR-767-3p	1.46	Higher	0.02444
hsa-miR-766-3p	1.23	Lower	0.03977	hsa-miR-29c-3p	1.46	Higher	0.01025
hsa-miR-342-5p	1.21	Lower	0.02444	hsa-miR-6737-3p	1.40	Higher	0.01614
hsa-miR-550a-3-5p	1.20	Lower	0.03485	hsa-miR-5739	1.39	Higher	0.01648
hsa-miR-324-3p	1.20	Lower	0.02585	hsa-miR-4787-5p	1.37	Higher	0.00309
				hsa-miR-3162-3p	1.37	Higher	0.03485
				hsa-miR-6069	1.36	Higher	0.02777
				hsa-miR-18b-3p	1.34	Higher	0.03001
				hsa-miR-6749-5p	1.32	Higher	0.00288
				hsa-miR-4313	1.31	Higher	0.03418
				hsa-miR-210-3p	1.23	Higher	0.04427

*An adjusted unpaired Student's t-test was used to calculate the P-value*.

*TGA-RV, transposition of the great arteries with a systemic right ventricle; HVs, healthy controls*.

**Table 3:** Correlation analysis for worsening HF (Spearman rank correlation).

**MicroRNA**	**Correlation coefficient (*r*)**	***P-*value**
miR-99a-5p	0.421	0.011
miR-423-3p	0.411	0.013
miR-183-3p	0.411	0.013
miR-6513-3p	0.392	0.018
miR-128-3p	0.363	0.029
miR-942-3p	0.363	0.030

### Validation by Single Real-Time RT-qPCR

**Figure 2:**
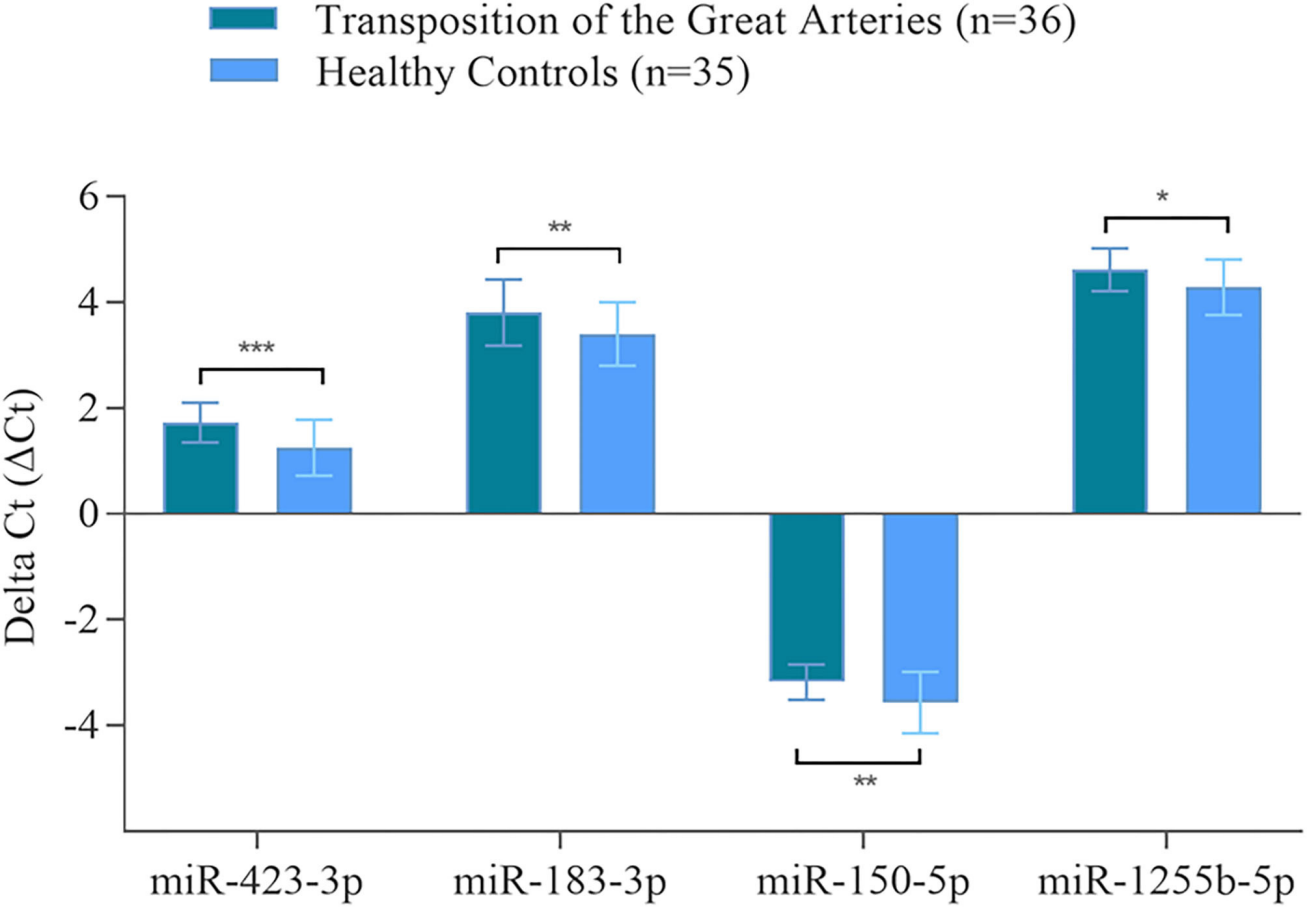
Validated miRNAs in the blood of TGA-RV patients compared to healthy controls as determined by RT-qPCR. Mean ΔCt of all TGA-RV patients and ΔCt of all healthy controls (lower ΔCt, higher abundance level). RNAU6B was used as endogenous control for normalization of miRNA. Unpaired Student's *t*-test and mean ± standard deviation were used to evaluate differences in abundance levels. *p ≤ 0.05; **p ≤ 0.01; ***p ≤ 0.001.

Using RT-qPCR, the validation of microarray analysis findings was performed for 21 differentially abundant miRNAs using the same samples which have been used for the microarray experiments. These 21 miRNAs were selected based on their highly significant differential abundance level in TGA-RV patients compared to that in the HVs as well as their significant relation to worsening HF according to microarray analysis. RT-qPCR showed the same direction of abundance changes as the microarray analysis for 19 out of 21 tested miRNAs, when comparing the samples from TGA-RV patients to HVs (Supplementary Figure 1). Significant changes in abundance level were confirmed for 11 miRNAs with lower abundance levels (namely miR-3200-3p, miR-128-3p, miR-133b, miR-423-3p, miR-150-5p, miR-1271-5p, miR-99a-5p, miR-550b-2-5p, miR-1285-3p, miR-1255b-5p, and miR-183-3p) and 2 miRNAs with higher abundance levels (namely miR-29b-3p and miR-19a-3p). Of the validated miRNAs, miR-423-3p, miR-183-3p, miR-150-5p and miR-1255b-5p were identified to be significantly associated with worsening HF and are illustrated in Figure 2.

### Predictors of Worsening HF

To evaluate predictors of worsening HF, Cox regression analysis was performed including the 4 miRNAs that were validated by RT-qPCR as well as NYHA class, the EF of the systemic RV and high sensitive troponin T (Table 4). In the multivariable analysis, EF of the systemic RV (*P* = 0.001), miR-183-3p (*P* = 0.001) as well as high sensitive troponin T (*P* = 0.002) turned out to be independent predictors of worsening HF.

**Table 4:** Predictors of worsening HF (Cox regression analysis).

**Variable**	**Univariable analysis** **HR (95% CI)**	***P-*value**	**Multivariable analysis** **HR (95% CI)**	***P-*value**
NYHA class	12.787 (2.757–59.308)	0.001	—	ns
EF of the systemic RV	0.898 (0.846–0.953)	<0.001	0.861 (0.745–0.995)	0.001
hs TNT	1.292 (1.071–1.559)	0.008	2.932 (0.714–12.043)	0.002
miR-423-3p	0.007 (0.000–1.113)	0.055	—	ns
miR-183-3p	0.194 (0.028–1.336)	0.096	0.005 (0.000–2.543)	0.001
miR-150-5p	7.527 (0.356–159.216)	0.195	Not included	—
miR-1255b-5p	2.106 (0.339–13.096)	0.424	Not included	—

*HR, hazard ratio; CI, confidence interval; NYHA, New York Heart Association; EF, ejection fraction; RV, right ventricle; hs TNT, high sensitive troponin T; ns, not significant*.

## Discussion

The present study aimed to analyze miRNA signatures in patients with TGA and a systemic right ventricle in order to identify those miRNAs that were associated with worsening HF. It is known that a right ventricle in the systemic position is prone to maladaptive remodeling and heart failure (5, 29, 30). Thus, it is important to identify those patients who are at risk for worsening or overt HF since it has tremendous implications on prognosis (3, 8). In TGA-RV patients, a NT-proBNP level exceeding 1000 pg/ml seems to be an indicator of worse prognosis (31, 32); therefore, the rise of NT-proBNP levels above this cut-off value, as well as the occurrence of overt HF, were used to define worsening HF in our cohort of patients.

In our study, the EF of the systemic RV, miR-183-3p and high sensitive troponin T were identified as independent predictors of worsening HF. It was indeed surprising that the EF of the systemic RV was predictive of worsening HF instead of NYHA class that is known to be a strong prognostic indicator in HF despite its subjective aspect (33). Our findings may be due to the fact that most of the patients with a severely reduced EF of the systemic RV were not severely limited in daily life activities or had only moderate heart failure symptoms. MiR-183-3p has been shown to discriminate non-HF-controls from patients with HFrEF but also HFpEF (34). Moreover, miR-183-3p has been found to be down-regulated in rats with chronic systolic HF and it was shown that treatment of these rats with vagus nerve stimulation resulted in an up-regulation of miR-183-3p along with lower NT-proBNP levels (35). In that study, Bcl-2 interacting protein 3 like was identified as the target gene of miR-183-3p. That study also indicates that miR-183-3p seems to be involved in the progression of HF and can be influenced by therapeutic interventions. Our results are different from the study results by Lai et al. identifying miR-18a and miR-486-5p to be negatively related to ventricular myocardial contractility in TGA patients after atrial switch operation using isovolumetric acceleration time to assess the systolic function of the systemic RV (23). However, it is known that isovolumetric acceleration time is derived from tissue Doppler measurements reflecting subtle changes of myocardial contractility. In contrast, in our study, clinical and prognostic parameters were used for the evaluation of miRNA signatures in order to identify miRNAs reflecting advanced heart disease and yielding prognostic impact. Thus, it is not surprising that the miRNAs identified in both studies are different because miRNA expression patterns might be different at the onset, during progression, in the acute or terminal stage of HF (21, 36).

Interestingly, miR-150-5p expression was not highly abundant in our cohort of patients. MiR-150 is known to regulate cardiac fibrosis and has been shown to be associated with atrial fibrillation and advanced left heart failure thus reflecting atrial and/or ventricular remodeling (37–39). In patients with univentricular heart, lower expression levels of miR-150-5p were found in patients prior to the occurrence of overt HF indicating its prognostic value (15). However, in our TGA-RV cohort, miR-150-5p was only weakly related to worsening HF and failed to be an independent predictor what may be due to the fact that heart failure was not as severe as in the univentricular heart cohort. These findings are in line with two studies in patients with atrial fibrillation indicating miR-150 levels to be lower in patients with persistent than paroxysmal AF thus reflecting more advanced atrial remodeling (40) and miR-183 to be one of the miRNAs that may indicate recurrence of paroxysmal AF in patients after pulmonary vein isolation thus reflecting more subtle changes in atrial remodeling (41). Although TGA-RV patients carry a high atrial arrhythmic burden that was also present in our study cohort, these specific atrial arrhythmias are a different entity compared to atrial fibrillation and usually occur recurrently and a long time before the presence of overt HF. Although they have been proposed as a surrogate parameter for right ventricular dysfunction and clinical outcome in TGA patients after atrial switch operation (42, 43), neither miR-183-3p nor miR-150-5p were associated with the presence of these specific atrial arrhythmias in our study population. This may be due to the fact that a substantial proportion of patients (>50%) experienced these specific atrial arrhythmias without developing overt or worsening HF during follow-up and that the follow-up period was probably too short.

Our study has several limitations that need to be addressed. The strengths of the present study include the large number of miRNAs screened in a TGA-RV patient group in order to identify miRNAs that predict worsening HF. To our knowledge, no such data is currently available in the literature. The small number of patients enrolled is certainly a criticism, however, TGA is a rare congenital heart defect and atrial switch operation has been replaced by the arterial switch procedure thus reducing the number of TGA-RV patients. Moreover, in the present study, the discriminative or prognostic power of the identified miR-183-3p in direct comparison to NT-proBNP cannot be assessed due to the study design and the use of NT-proBNP levels for the definition of worsening HF. Thus, our findings should be confirmed in a larger study cohort comprising more patients with overt HF.

## Conclusion

In patients with TGA-RV, ejection fraction of the systemic RV, miR-183-3p and high sensitive troponin T were found to be independent predictors of worsening HF thus yielding prognostic impact. Since miR-183-3p seems to be involved in disease progression, it might be used as an additional biomarker in the risk assessment of these patients.

## Data Availability Statement

The datasets presented in this study can be found in online repositories. The names of the repository/repositories and accession number(s) can be found below: GEO, GSE179105.

## Ethics Statement

The studies involving human participants were reviewed and approved by Institutional Review Board (No. 73/09). Written informed consent to participate in this study was provided by the participants' legal guardian/next of kin.

## Author Contributions

MA-H: performed experimental work, particularly the miRNA isolation, array experiment, and RT-qPCR validation. MA-H and TR-H: manuscript writing and statistical analysis. TR-H, HA-K, and EM: designed the study, coordinated the molecular biology experiment, and edited the manuscript. TR-H and HA-K: recruited and examined controls, diagnosed patients, and collected blood samples. MA-H, TR-H, EM, and HA-K: supervision, project administration, and funding acquisition. All authors read and approved the final manuscript.

## Funding

This work was supported by the German Heart Foundation, Frankfurt/Main, Germany, for the research on adults with congenital heart disease and the Hedwig Stalter Foundation.

## Conflict of Interest

The authors declare that the research was conducted in the absence of any commercial or financial relationships that could be construed as a potential conflict of interest.

## Publisher's Note

All claims expressed in this article are solely those of the authors and do not necessarily represent those of their affiliated organizations, or those of the publisher, the editors and the reviewers. Any product that may be evaluated in this article, or claim that may be made by its manufacturer, is not guaranteed or endorsed by the publisher.
