# Supplementary figures and images for: MicroRNA-183-3p Is a Predictor of Worsening Heart Failure in Adult Patients With Transposition of the Great Arteries and a Systemic Right Ventricle
Source: Front Cardiovasc Med. 2021 Sep 8;8:730364. doi: 10.3389/fcvm.2021.730364 (PMC8455927; doi:10.3389/fcvm.2021.730364)

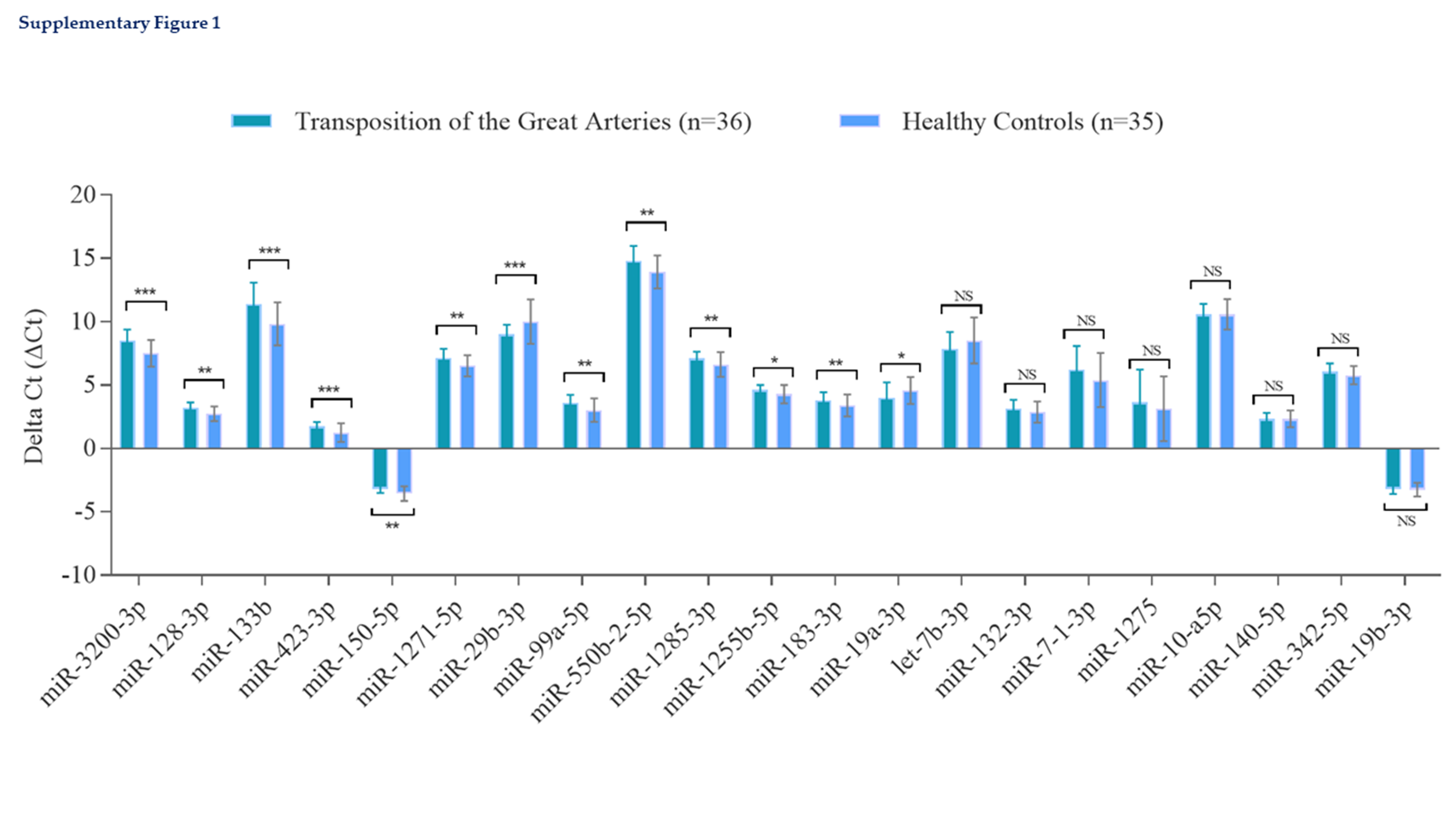

Supplement: Supplementary file 1 [file Image_1.TIF]
